# Targeting PCNA/PARP1 axis inhibits the malignant progression of hepatocellular carcinoma

**DOI:** 10.3389/fphar.2025.1571786

**Published:** 2025-04-17

**Authors:** Jipin Li, Tao Yong, Yali Chen, Tingyu Zeng, Kaifeng Zhang, Shuping Wang, Youcheng Zhang

**Affiliations:** ^1^ Department of General Surgery, The Second Hospital of Lanzhou University, Lanzhou, China; ^2^ Key Laboratory of Preclinical Study for New Drugs of Gansu Province, Institute of Biochemistry and Molecular Biology, School of Basic Medical Sciences, Lanzhou University, Lanzhou, China

**Keywords:** hepatocellular carcinoma, PCNA, PARP1, DNA damage repair, cell cycle progression

## Abstract

**Introduction:**

Proliferating cell nuclear antigen (PCNA) is associated with the proliferation and recurrence of various cancers, and its high expression is associated with poor prognosis in hepatocellular carcinoma (HCC) patients. However, the mechanistic role of PCNA in HCC progression remains poorly understood. This study aimed to investigate how PCNA regulates DNA damage repair and cell cycle progression in HCC, with a focus on its interaction with poly (ADP-ribose) polymerase 1 (PARP1) and therapeutic implications.

**Methods:**

PCNA was targeted genetically and pharmacologically in HCC cells to assess its effects on DNA damage repair and cell cycle arrest. Protein-protein interactions between PCNA and PARP1 were validated through co-immunoprecipitation and functional assays. The sensitivity of HCC cells to the PARP1 inhibitor Olaparib was evaluated under PCNA inhibition. Synergistic effects of AOH1160 (a PCNA inhibitor) and Olaparib were tested in vitro and in vivo using proliferation assays, DNA damage quantification, and cell cycle analysis. Prognostic relevance of PCNA expression was analyzed using TCGA datasets.

**Results:**

Targeting PCNA suppressed DNA damage repair and induced cell cycle arrest in HCC cells. Mechanistically, PARP1 was identified as a downstream target of PCNA and directly interacted with PCNA. Inhibiting the expression or activity of PCNA increased the sensitivity of HCC cells to the PARP1 inhibitor, Olaparib. In addition, AOH1160 and Olaparib synergistically inhibited the proliferation, DNA damage repair and cell cycle progression of HCC cells. Elevated PCNA levels correlated with unfavorable HCC prognosis, supporting its role as a therapeutic biomarker. In vivo experiments also confirmed that repression of the PCNA/PARP1 axis significantly reduced HCC tumor growth.

**Discussion:**

This study elucidates the relationship between PCNA and PARP1 in regulating the malignant progression of HCC, and highlight the pivotal role of PCNA/PARP1 axis in DNA damage repair and cell cycle progression. The correlation between elevated PCNA levels and unfavorable prognosis underscores its potential as a therapeutic biomarker. Repression of PCNA/PARP1 axis significantly inhibits the malignant proliferation of HCC cells both in vitro and in vivo. Collectively, the study provides a mechanistic foundation for therapies targeting PCNA/PARP1 axis.

## 1 Introduction

Hepatocellular carcinoma (HCC) is a common malignancy and one of the leading causes of cancer-related death worldwide (Dopazo et al., 2024; Sankar et al., 2024). In particular, the incidence of HCC in China accounts for over 50% of the global total (Rumgay et al., 2022). Because the subtle early symptoms of HCC, many patients are diagnosed at an advanced stage. At this stage, HCC shows significant invasiveness and metastatic potential, resulting in an extremely high mortality rate, making it become one of the most difficult malignancies to treat (Dopazo et al., 2024; Sankar et al., 2024; Yang et al., 2024). Surgical resection is currently the most effective curative treatment for HCC patients with localized lesions, however, it is not available for more than 50% of HCC at advanced stage (Vogel et al., 2022; Brown et al., 2023). Systematic chemotherapy has become the main treatment for advanced HCC. Besides, there have been significant advances in the systemic treatment options for HCC over the past few decades, with several approvals of immune checkpoint inhibitors and tyrosine kinase inhibitors in patients with HCC (Rimassa et al., 2023; Sankar et al., 2024). Despite the emergence of new systemic therapies, survival for patients with advanced HCC remains poor, most patients with advanced HCC still suffer from therapeutic resistance and disease malignant progression (Brown et al., 2023; Lei et al., 2024). Meanwhile, the side effects of systemic chemotherapy seriously affect the life quality of patients. With the development of sequencing technology and bioinformatics, novel targets and pathways driving the malignant progression of HCC have been rapidly discovered (Yang et al., 2024; Suresh et al., 2023). Therefore, identification of novel targets and discovery of innovative targeted therapies have potential to improve survival and quality of life for patients with challenging HCC.

Proliferating cell nuclear antigen (PCNA) is a ring-shaped homotrimer protein, and it is evolutionarily well conserved found in all eukaryotic species from yeast to humans, reflecting its essential role in cellular processes (Cardano et al., 2020). The overall structure of PCNA resembles a sliding clamp around DNA, which is crucial for its role in DNA replication (Arbel et al., 2021; Shao et al., 2023). PCNA serves as a processivity factor for DNA polymerase and is regulated by cell cycle checkpoints to maintain genomic integrity and prevent the propagation of DNA errors (Cazzalini et al., 2003). PCNA is not only involved in DNA replication, but also in other vital cellular processes such as DNA damage repair, chromatin remodeling, and cell cycle progression (Boehm et al., 2016). Mechanistically, PCNA interacts with its binding partner PCNA clamp-associated factor (PCLAF/PAF15/KIAA0101) to stabilize the trimeric conformation of PCNA, thereby facilitating the recruitment of DNA polymerases to replication forks and ensuring efficient DNA synthesis (Xie et al., 2024). Beyond replication, the PCNA-PCLAF complex orchestrates DNA repair pathway selection and modulates cell cycle checkpoints, particularly under genotoxic stress (Kim B. et al., 2024). Notably, PCNA is frequently overexpressed in highly proliferating tumor cells, where it functions as both a biomarker of uncontrolled proliferation and a promising therapeutic target. Elevated PCNA levels are associated with poor prognosis in various cancers (Suresh et al., 2023). Emerging evidence indicates that PCNA drives the malignant progression of cancer through modulating various pathophysiological processes. For instance, phosphorylation of PCNA at tyrosine 211 (Y211) has been shown to promote metastatic dissemination and sustain cancer stemness, highlighting its role in tumor evolution (Wang et al., 2022). Meanwhile, PCNA on cell surface can enhance immune evasion by prevention of natural killer cells activation and degranulation through the inhibitory receptor NKp44 (Kundu et al., 2019; Knaneh et al., 2023). Furthermore, PCNA participates in the DNA damage repair of various tumors by regulating or interacting with other factors such as nuclear insulin-like growth factor 1 receptor (IGF1R) and a nuclease scaffold SLX4 (Waraky et al., 2017; Yang et al., 2020; Kim S. et al., 2024). This suggests that targeted inhibition of PCNA expression or activity may be an effective strategy to inhibit cancer cell proliferation (Kwan et al., 2024). Previous studies have shown that PCNA is overexpressed in HCC tissues compared to normal liver tissues, and is associated with aggressive tumor behavior, poor differentiation, and unfavorable clinical outcomes, suggesting that PCNA may be a potential target for the treatment of HCC (Cheng et al., 2020; Shen et al., 2022).

In recent years, targeted DNA damage response network strategies have achieved remarkable results in the treatment of cancer (Groelly et al., 2023). Indubitability, poly (ADP-ribose) polymerase (PARP) inhibitors represented by Olaparib are the most successful representatives and have been used in the clinical treatment of breast cancer, ovarian cancer, prostate cancer, colon cancer, pancreatic cancer, and other cancers (Morganti et al., 2024; Zeng et al., 2024). Similar to PCNA, PARP1 is another important DNA repair signaling molecule that is overexpressed in many cancers and associated with poor prognosis. PARP1 plays a crucial role in DNA single-strand breaks (SSBs) repair through the base excision repair (BER) pathway (Wei et al., 2024; Laspata et al., 2024). When the expression or enzymatic activity of PARP1 is inhibited, SSBs accumulate and eventually lead to double-strand breaks (DSBs) during DNA replication. If the homologous recombination (HR) repair pathway, the precise DNA DSBs repair pathway, is not activated at this time, it will lead to a large accumulation of DNA fragments, resulting in chromosome instability and cell death (Wei et al., 2024; Zhang and Zha, 2024; Laspata et al., 2023). Based on synthetic lethality, PARP inhibitors have been widely used in the treatment of cancers with HR deficiency. However, only a small portion of cancers exhibit HR deficiency, and the majority of cancer patients still do not benefit from PARP inhibitors (Li et al., 2020). Meanwhile, the issue of resistance to PARP inhibitors also limits its clinical application (Dilmac and Ozpolat, 2023). PCNA is the center of DNA replication and DNA damage repair and plays an important role in coordinating the function of protein factors such as polymerase δ (Cazzalini et al., 2003). Importantly, the repair of DNA damage is tightly regulated by cell cycle checkpoints, which act as surveillance mechanisms to ensure genomic stability. In response to DNA damage, key checkpoint kinases such as ATM (ataxia-telangiectasia mutated) and ATR (ataxia-telangiectasia and Rad3-related) activate downstream effectors, including CHK1 and CHK2, to halt cell cycle progression and facilitate repair (Smith et al., 2020). If damage is beyond repair, these pathways can induce apoptosis or senescence to prevent malignant transformation. Given that both PCNA and PARP1 are closely linked to DNA replication stress and repair, investigating the molecular mechanism of PCNA in regulating DNA damage repair and the regulatory relationship between PCNA and PARP1 can not only provide a novel strategy for targeted therapy of HCC but also expand the clinical indication of PARP inhibitors.

Herein, we delineate the mechanistic interplay between PCNA and PARP1 in HCC progression and therapeutic resistance. We demonstrate that PCNA directly interacts with PARP1 to sustain DNA repair proficiency and cell cycle progression. Genetic or pharmacological PCNA inhibition sensitizes HCC cells to Olaparib by impairing compensatory DDR pathways. Furthermore, the PCNA inhibitor AOH1160 synergizes with Olaparib to suppress HCC growth *in vitro* and *in vivo*. Our findings establish the PCNA/PARP1 axis as a key regulator of HCC malignancy and provide theoretical support for combining PCNA inhibitors with PARP inhibitors in HCC treatment, as well as for the development of dual-target PCNA/PARP1 inhibitors.

## 2 Materials and methods

### 2.1 Reagents, primers, and antibodies

AOH1160, a PCNA inhibitor, which targets amino acids region L126-Y133 of PCNA (Gu et al., 2018), and Olaparib (AZD2281), a PARP inhibitor, were purchased from Targetmol (Wellesley Hills, MA, United States) and dissolved in dimethyl sulfoxide (DMSO) at 10 mM to produce stock solutions at −20°C. All other chemicals used were analytical grade without purification. The antibodies are listed in Supplementary Table 1.

### 2.2 Bioinformatics analysis

The differential expression of genes in the cancer genome atlas (TCGA) database was analyzed by Gene Expression Profiling Interactive Analysis (GEPIA) online website. The mRNA expression of genes in cancer samples were compared with that in normal adjacent from TCGA database, and the P value was obtained by one-way ANOVA. The significant values of P-value and folding change are 0.05 and 2.0 respectively. The correlation between gene and patient survival was evaluated using the Kaplan-Meier Plotter. Samples were divided into groups with high and low expression according to the median expression. The 95% confidence interval, log-rank risk ratio (HR), and P value of overall survival (OS) and disease-free survival (DFS) were analyzed. The relationship between gene expression and prognosis was analyzed using receiver operating characteristic (ROC) curves. The original clinical data source of 424 HCC RNA sequencing information was obtained from the TCGA database. The R package pROC analyzed the area under the curve (AUC) values, and the data were visualized as ggplot2. The AUC value greater than 0.9 indicates strong evidence of model success (Zhang et al., 2021). Statistical analysis and visualization were performed in R v4.0.3. The expression correlation of genes was performed using TCGA data in the GEPIA online tool. Immunohistochemical results for the differential expression of PCNA and PARP1 in HCC tissues and normal adjacent tissues were obtained from the Human Protein Atlas (HPA) online database (https://www.proteinatlas.org/). The protein-protein interaction (PPI) network of PCNA was constructed using the Search Tool for the Retrieval of Interacting Genes/Proteins (STRING) protein-protein interaction networks functional enrichment online platform (https://cn.string-db.org/).

### 2.3 Cell culture

The human HCC cell lines HepG2 and Hep3B cell lines were procured from Cell Resources Center of Shanghai Academy of Life Sciences (Shanghai, China). Huh7 cell lines were purchased from Procell Life Science & Technology Co., Ltd. (Wuhan, China). HepG2 cells, Hep3B cells, and Huh7 cells were cultured in DMEM (KGL1206, KeyGEN Biotech, Nanjing, Jiangsu, China) with 10% fetal bovine serum (FBS). All cells were incubated at 37°C in a 5% CO_2_ atmosphere. The cells were not passaged more than six times from collection to use and were authenticated by STR profiling regularly every half year.

### 2.4 Lentivirus transfection

Lentiviral recombination vectors of human PCNA gene (pGV492-PCNA) and its scrambled control (pGV492-NC), Lentiviral recombination vectors of short-hairpin RNA against PCNA (pGV112-shPCNA), short-hairpin RNA against PARP1 (pGV248-shPARP1) and the scrambled control (pGV112-shNC) were constructed and purchased from Genechem Co. Ltd. (Shanghai, China). The pGV112-shPCNA vector and pGV248-shPARP1 vector were confirmed by sequencing. HepG2 cells and Huh7 cells were infected with oePCNA, shPCNA, shPARP1 and shNC Lentiviral vectors using HitransG A promoting reagent according to the manufacturer’s instructions. Hep3B cells were infected with oePCNA and oeNC. After infection with lentiviral vector for 3 days, culture medium containing virus was removed. Transfected cells were allowed for growth for 3–5 days, and then treated with 2.0 μg/mL puromycin for 24 h to select positive infected cells. For the rescue experiment, cells were infected with one lentiviral vector for 3 days and the culture medium containing virus was removed. The cells were then infected with another vector for 3 days, and the culture medium containing virus was removed. Cells transfected with two vectors were allowed for growth for 3–5 days, and then treated with 2.0 μg/mL puromycin for 24 h to select positive infected cells. All transfected cells were validated by quantitative PCR and Western blots, and maintained in a medium containing 1.0 μg/mL puromycin. The target sequence of shPCNA is 5′-AAG​CCA​CTC​CAC​TCT​CTT​CAA-3′, target sequence of shPARP1 is 5′-CAA​CTC​CAG​GAA​GGA​AAC​CAA-3′, target sequence of shNC is 5′-TTC​TCC​GAA​CGT​GTC​ACG​T-3’.

### 2.5 RNA sequencing and data processing of DEGs

According to the manufacturer’s manual, total RNA was extracted from the HepG2 cells transfected with shNC or shPCNA using TRIzol reagent (Vazyme, Nanjing, China). A total of 500 ng of RNA was used to prepare libraries using the NEBNext Ultra RNA Library Prep Kit for Illumina. RNA quantity and quality were assessed on an Agilent 2,100 Bioanalyzer. RNA library sequencing was performed on an Illumina HiSeqTM 2,500/4,000 by Gene *Denovo* Biotechnology Co., Ltd. (Guangzhou, Guangdong, China). Differentially expressed genes (DEGs) in the shNC group vs. the shPCNA group were identified based on a |log2FC| > 1.0 and an adjusted P < 0.05. DEGs with a |log2FC| < 1.0 were considered downregulated genes, while DEGs with a |log2FC| > 1.0 were considered upregulated genes (Wang et al., 2021).

### 2.6 GO, KEGG pathway, reactome and GSEA enrichment analysis

The biological attributes of the DEGs were identified using gene ontology (GO) enrichment and gene set enrichment analysis (GSEA) enrichment analysis. The functional attributes of the DEGs were identified using Kyoto Encyclopedia of Genes and Genomes (KEGG) pathway enrichment, Reactome enrichment and GSEA enrichment analysis. GO enrichment analysis, KEGG pathway enrichment analysis, Reactome enrichment, and GSEA enrichment analysis were performed using Omicsmart online platform (http://www.omicsmart.com) (Huang et al., 2022).

### 2.7 Colony formation assay

For colony formation analysis, single-cell suspensions were seeded at 1 × 10^3^ cells/well into 24-well plates. After overnight incubation, the cells were treated with the designed drugs for 10–14  days, and the medium was replaced every 3 days. The colonies were fixed in formaldehyde and stained with 0.1% crystal violet (Solarbio Life Sciences, China), and imaged using a fully automated live cell imaging system (Thermo Fisher EVOS M7000, Waltham, MA, United States). The quantitative analysis on the results of the colony formation assay was performed by ImageJ software (Version 1.53K).

### 2.8 Cell viability assay and determination of drug synergy

The cells of interest (1.5 × 10^3^–4 × 10^3^ cells per well) were seeded into 96-well plates overnight in 100 μL of complete growth medium and then treated with AOH1160 and Olaparib at different combination ratios for 6 days in triplicate. Following treatments, MTT solution was added to each well, plates were incubated for 4 h at 37°C, medium was removed, and formazan crystals were dissolved in DMSO. Cell viability was evaluated by measuring the well absorbances at 490 nm using microplate reader (Synergy NEO2, BioTek). The combined effects of AOH1160 and Olaparib were assessed using Calcusyn software (Biosoft, Cambridge, UK). Combination indexes (CI), which were used to evaluate the effects of two-drug combinations, were calculated using the Chou-Talalay method. Drug synergism was defined as a CI value of <1, while antagonism was defined as a value of >1. Additivity was defined as a CI = 1 (Chou, 2006; Wu et al., 2022).

### 2.9 Analysis of apoptosis and cell cycle

Flow cytometry analysis was used to assess cell apoptosis and the cell cycle (Huang et al., 2022). The cells of interest were treated with the designed drugs for 6 days and digested with EDTA-free trypsin. For apoptosis evaluation, the cells were collected and stained using an Annexin V-FITC/PI apoptosis kit (AT101, MultiSciences, Hangzhou, China) or Annexin V-PE/7-AAD apoptosis kit (AT104, Multisciences, Hangzhou, China). The cell cycle was analyzed by a PI cell cycle detection kit (CCS012, MultiSciences, Hangzhou, China). The above cells were all identified and quantified by a flow cytometer (NovoCyte Quanteon, United States) according to the manufacturer’s instructions, and the data were analyzed by FlowJo v10 software, and the cell cycle data were analyzed by ModFit LT5.

### 2.10 Alkaline comet assay

Alkaline comet assay was performed as previously reported (Wu et al., 2022; Walsh and Kato, 2023). Briefly speaking, HepG2 cells or Huh7 cells were treated with different concentrations of AOH1160, Olaparib and their combination for 6 days. After treatment, 1✕10^4^ cells were mixed with low melting point agarose at a ratio of 1:10 (v/v), layered onto the slide, lysed by the lysis buffer at 4°C for 2 h, and unwound by alkaline unwinding solution for 30 min at room temperature. The gel electrophoresis was conducted in the condition of 25V and 40 min. The DNA was finally stained with propidium iodide (PI) for 15 min at room temperature. Then the slides were observed and imaged under a fluorescence microscope (Nikon-ECLIPSE 80i, Tokyo, Japan). Subsequently, we employed the CASP software (Version 1.2.3) to quantify the percentage of tail DNA measured in the comet assay (Collins et al., 2023).

### 2.11 Immunoblotting (IB) and immunoprecipitation (IP) assays

The western blot protocol has been described in detail previously (Wang et al., 2021; Wu et al., 2022). In short, HepG2 cells were lysed in RIPA cell-lysis buffer (KGB5203-100, KeyGEN Biotech, Nanjing, China) containing protease and phosphatase inhibitors on ice for 30 min. The lysates were centrifuged at 12,000 rpm for 15 min, the supernatants were collected, and the protein concentrations were determined with a Bicinchoninic (BCA) Protein Assay Kit (Solarbio, PC0020). A total of 20–30 μg of protein was separated on 8%–15% SDS-PAGE gels and transferred to PVDF membranes (Millipore, United States, IPVH00010, ISEQ00010). Membranes with protein were blocked with 5% (w/v) skim milk, incubated with primary antibody overnight at 4°C, and then incubated with secondary antibodies (1:5,000) for detection. The primary and secondary antibodies are described in Supplementary Table 1. The immunoprecipitation (IP) assay was performed using Protein A/G Magnetic IP/Co-IP kit (ACE Biotechnology, Nanjing, China). Briefly, the cells were lysed in enhanced lysis buffer containing protease and phosphatase inhibitors. The supernatants were incubated with 1–4 μg of primary antibodies overnight at 4°C on a rotating platform, followed by immunoblotting analysis. ImageJ was used to quantify the immunoblotting results by measuring the protein band densities.

### 2.12 RNA extraction and Q-PCR

Total RNA was extracted with TRIzol reagent (Vazyme, Nanjing, Jiangsu, China) according to the manufacturer’s instructions. RNA was reverse transcribed into cDNA by using a HiScript II one-step RT-PCR kit (P612-01, Vazyme, Nanjing, China) with 1.0 μg of total RNA in a 20 μL reaction system. 1.0 μL of the resulting cDNA was used in per quantitative PCR (Q-PCR) in triplicate. Q-PCR was carried out using ChamQ SYBR Q-PCR master mix (Q311–02, Vazyme, Nanjing, China) on a QuantStudio three real-time PCR detection system (Life Tech, New York, United States). Relative expression levels were calculated as ratios normalized against the endogenous control (GAPDH). The relative fold changes of candidate genes were analyzed using the 2^−ΔΔCT^ method. All primers were synthesized by Tsingke Biotech (Wu et al., 2022). The sequences of the primers are listed in Supplementary Table 2.

### 2.13 Immunofluorescence assay

After treatment, cells (5 × 10^4^) were seeded on a confocal plate. After overnight incubation, cells were treated as described in the text. Cells were then collected and fixed with 4% formaldehyde, permeabilized with 0.1% (v/v) Triton X-100 in PBS, and blocked with 1% (w/v) BSA in PBS for 1.0 h. After blocking, cells were incubated with the phospho-Histone H2A.X (Ser139) rabbit antibody at 4°C overnight, washed twice with PBS, and incubated for 2.0 h at room temperature with appropriate secondary antibodies. Cells were counterstained with DAPI (4′,6-diamidine-2′-phenylindole dihydrochloride). Fluorescence signals were visualized using a Zeiss LSM 900 laser scanning confocal microscope (Jena, Germany) and photographs were taken at a magnification of ×40 (Wang et al., 2021; Wu et al., 2022). The γ-H2AX foci in each cell were captured and counted.

### 2.14 Mouse xenograft tumor model

All animal procedures were carried out following the institutional guidelines for the care and use of laboratory animals and approved by the Committee on the Ethics of Animal Experiments of Lanzhou University (Lzujcyxy20230314). Every effort was made to ensure the comfort and safety of the animals. Female BALB/c nude mice aged 6–8 weeks were randomly used to establish xenograft models. 5 × 10^6^ HepG2 cells (wild-type, empty control vector-transfected, stably PCNA-knockdown vector transfected) were subcutaneously inoculated into the right axilla of the mice. After the average tumor volume (mm^3^) reached to 50mm^3^, the tumor-bearing mice were treated using different strategies (n = 5/group). Olaparib (40 mg/kg), AOH1160 (20 mg/kg), the combined group of Olaparib (20 mg/kg) and AOH1160 (10 mg/kg), the sequential treatment (40 mg/kg Olaparib for 10 days, followed by 20 mg/kg AOH1160 for 10 days) were administered by intraperitoneal injection for 21 consecutive days. The sequential regimen was designed to model clinical scenarios where PARP inhibitor resistance emerges, thereby evaluating potential of AOH1160 to overcome acquired therapeutic resistance. Tumor volume was recorded every 2 days using a digital caliper. Tumor volume was calculated using the formula, (ab^2^)/2, where a and b represent the length and width of the tumor (Wu et al., 2022). For ethical considerations, mice were euthanized via CO_2_ inhalation after 28 days.

### 2.15 Histological staining

The xenografted tumor tissues for immunohistochemistry (IHC) staining and the lung, liver, heart, kidney and spleen tissue samples of HepG2 xenograft tumor model mice were fixed in 4% paraformaldehyde, embedded in paraffin and sectioned at 3-μm thickness. For IHC staining, the slides were incubated in primary antibody diluted in blocking solution overnight at 4 °C. After incubation peroxidase conjugated secondary antibody was used against the primary antibody. For chromogenic detection, 3,3′-diaminobenzidine tetrahydrochloride (DAB) (Sigma, United States) was used as the substrate for peroxidase. Slides were counterstained with hematoxylin. Cells with brown nuclei were considered as positively stained. For haematoxylin and eosin (H&E) staining, slides were stained with Mayer’s haematoxylin and 0.1% sodium bicarbonate and counterstained with Eosin Y solution (Beyotime, Shanghai, China). Each group of samples was observed with Nikon-ECLIPSE 80i microscope with a Nikon DS-Ri2 Digital Camera (Tokyo, Japan).

### 2.16 Statistics

All data were representative of three independent experiments and illustrated as means ± standard error of the mean. Differences between groups were analyzed by one-way or repeated measures ANOVA using SPSS Version 20.0 software (SPSS Inc., Chicago, IL). P < 0.05 was considered statistically significant.

## 3 Results

### 3.1 PCNA promotes the malignant proliferation of HCC cells

**FIGURE 1:**
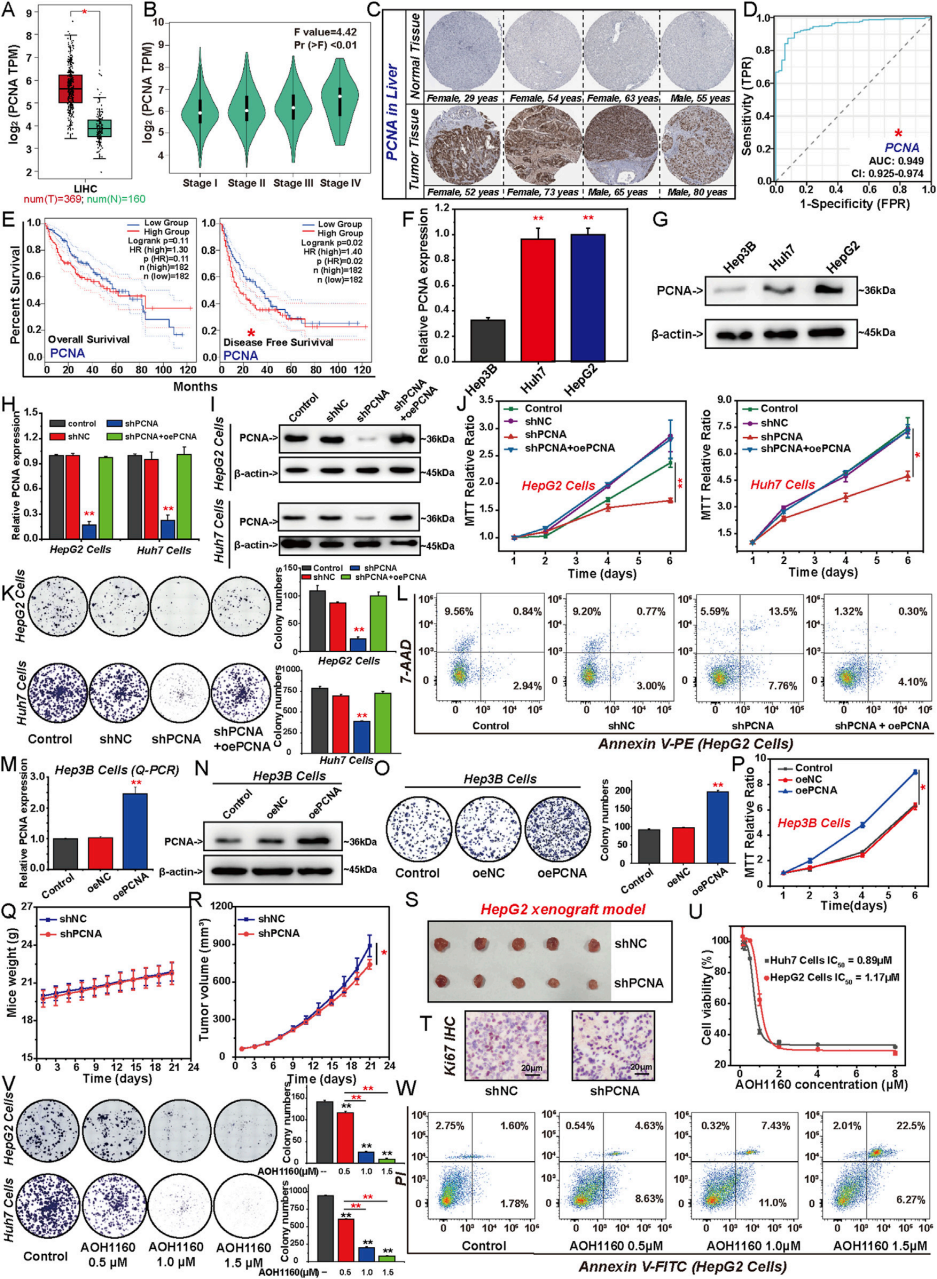
PCNA promotes the malignant proliferation of HCC cells. The differential expression of PCNA between HCC tissues and adjacent tissues obtained from TCGA dataset **(A, B)** and the HPA databases **(C)**. **(D)** Diagnostic ROC for PCNA in HCC and normal samples. **(E)** The impact of PCNA mRNA expression on patient survival was analyzed by Kaplan-Meier survival curve. The expression levels of PCNA in HCC cell lines were detected via Q-PCR **(F)** and Western blotting **(G)**. HepG2 cells and Huh7 cells were transfected by shNC, shPCNA, shPCNA and oePCNA, the transfected efficiency was verified by Q-PCR **(H)** and Western blotting **(I)**. The viability and colongenic growth of HepG2 cells and Huh7 cells with or without PCNA knockdown was analyzed using MTT assay **(J)** and colony formation assay, followed by quantification of colony numbers. **(K)**. **(L)** Apoptosis of HepG2 cells with or without PCNA knockdown was assessed via flow cytometry with 7-AAD and annexin V-PE double staining. Hep3B cells was transfected by oeNC and oePCNA, the transfected efficiency was verified by Q-PCR **(M)** and Western blotting **(N)**. The viability and colongenic growth of Hep3B cells with or without PCNA overexpression was analyzed using colony formation assay, followed by quantification of colony numbers. **(O)** and MTT assay **(P)**. **(Q–S)** HepG2 cells were subcutaneously implanted into nude mice after shNC or shPCNA transfection. Mice weight **(Q)** and tumor volume **(R)** were measured. The picture shows the size of the tumor **(S)**. **(T)** Immunohistochemical staining of Ki67 in the isolated xenograft tumor. **(U)** The IC_50_ of AOH1160, PCNA inhibitor, in HepG2 and Huh7 cells. **(V)** Colony formation of HepG2 cells and Huh7 cells treated with different concentrations of AOH1160, followed by quantification of colony numbers. **(W)** Apoptosis of HepG2 cells treated with different concentrations of AOH1160. The results from three independent experiments were statistically analyzed using one-way ANOVA: *P < 0.05, **P < 0.01.

We evaluated the differential expression of PCNA in HCC tissues and adjacent normal tissues from TCGA and HPA databases. As depicted in Figures 1A–C, the expression of PCNA was significantly higher in HCC tissues compared to normal liver tissues and its expression levels increased with the progression of tumor stages. The ROC curve analysis for PCNA revealed an AUC value of 0.949, indicating that PCNA expression was significantly associated with poor prognosis of HCC (Figure 1D). The log-rank test analysis revealed that patients with HCC with lower PCNA expression had significantly longer survival than those with higher PCNA (Figure 1E). PCNA expression was highly expressed in HepG2 cells and Huh7 cells among three HCC cell lines (Figures 1F, G), so we selected them to investigate the effect of PCNA knockdown on the proliferation of HCC cells (Figures 1H, I). As shown in Figures 1J, K, PCNA knockdown inhibited proliferation and clonogenic growth, whereas overexpress of PCNA rescued these effects. PCNA silencing significantly promoted apoptosis in HepG2 cells (Figure 1L). To further explore the role of PCNA in HCC progression, we investigated its effect on Hep3B cells by inducing PCNA overexpression (Figures 1M, N). The result showed that PCNA overexpression enhanced Hep3B cells proliferation and clonogenic potential. Furthermore, we assessed the impact of PCNA knockdown on tumor growth in a HepG2 xenograft model using nude mice. Silencing PCNA significantly reduced tumor volume without affecting body weight and led to a notable decrease in Ki67 expression (Figure 1Q-T). AOH1160 is a small molecular inhibitor of PCNA identified through high-throughput screening, targeting a surface pocket partly delineated by the L126-Y133 region of PCNA (Gu et al., 2018). To evaluate its cytotoxic effects on HCC cells, HepG2 and Huh7 cells were treated with AOH1160. MTT assays revealed that AOH1160 exhibited potent anticancer effect in a dose-dependent manner on HCC cells (Figure 1U), with calculated half-maximal inhibitory concentration (IC_50_) values of 1.17 µM for HepG2 and 0.89 µM for Huh7 cells. Based on these results, AOH1160 was used at concentrations of 0.5 µM, 1.0 µM, and 1.5 µM in the subsequent assays. Similar to the genetic inhibition of PCNA, AOH1160 treatment significantly reduced HepG2 and Huh7 colony formation while also increasing apoptosis in HepG2 cells (Figures 1V, W).

### 3.2 PCNA regulates genes involved in DNA repair and cell cycle progression

**FIGURE 2:**
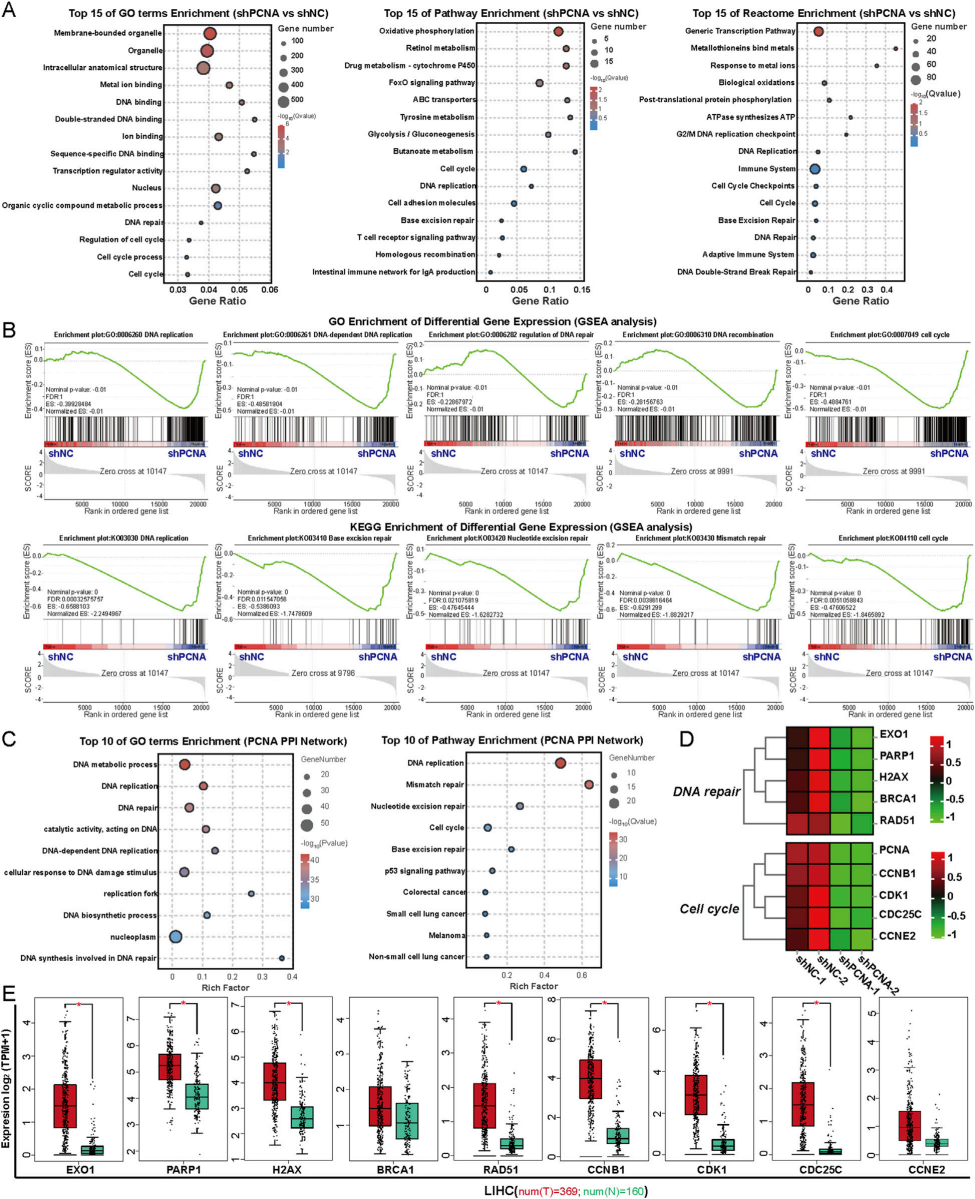
PCNA regulates genes involved in DNA repair and cell cycle progression. **(A)** Top 15 biological pathways of these downregulated genes after PCNA knockdown in HepG2 cells were analyzed by GO enrichment, KEGG pathway enrichment, and Reactome enrichment. **(B)** GSEA plots of the downregulated gene signature resulting from the knockdown of PCNA in HepG2 cells. **(C)** GO enrichment and KEGG pathway enrichment analysis of PCNA PPI network. **(D)** Heatmap plot of the key DEGs induced by PCNA knockdown. **(E)** The differential expression of the key DEGs in HCC of the TCGA project.

To elucidate the mechanism of PCNA in promoting the proliferation of HCC cells, we performed RNA sequencing (RNA-Seq) to analyze the effect of PCNA on the expression of total genes in HepG2 cells (Figures 2A–D). Knockdown of PCNA induced upregulation of 537 DEGs and downregulation of 156 DEGs in HepG2 cells. The results of GO functional enrichment, KEGG pathway enrichment and Reactome enrichment demonstrated that DEGs induced by knockdown of PCNA were mostly associated with DNA replication, DNA repair and cell cycle (Figure 2A). Supportively, the GSEA enrichment showed that high PCNA expression was positively correlated with the enrichment of gene sets related to DNA replication, DNA repair and cell cycle pathways (Figure 2B). Subsequently, we constructed the protein-protein interaction (PPI) network of PCNA, and analyzed the top 50 protein by the GO enrichment and KEGG pathway enrichment. The enrichment results showed that proteins in the PCNA PPI network were mainly involved in DNA replication, DNA repair, and the cell cycle (Figure 2C). And then, we selected the key genes-involved in DNA repair and cell cycle. Knockdown of PCNA led to significant downregulation of five genes-involved in DNA repair, exonuclease 1 (EXO1), PARP1, histone H2AX (H2AX), breast cancer susceptibility gene 1 (BRCA1), and recombinase Rad51 (RAD51), and four genes-involved in cell cycle progression, cyclin B1 (CCNB1), cyclin E2 (CCNE2), cyclin-dependent kinase 1 (CDK1), and cell-division cycle 25C (CDC25C) (Figure 2D). In addition, expression analysis of those key genes in HCC tissues compared to normal tissues demonstrated that EXO1, PARP1, H2AX, BRCA1, RAD51, CCNB1, CDK1, CDC25C, and CCNE2 were overexpressed in HCC tissues, further supporting the role of these genes in HCC progression (Figure 2E).

### 3.3 Repression of PCNA inhibits DNA repair in HCC cells

**FIGURE 3:**
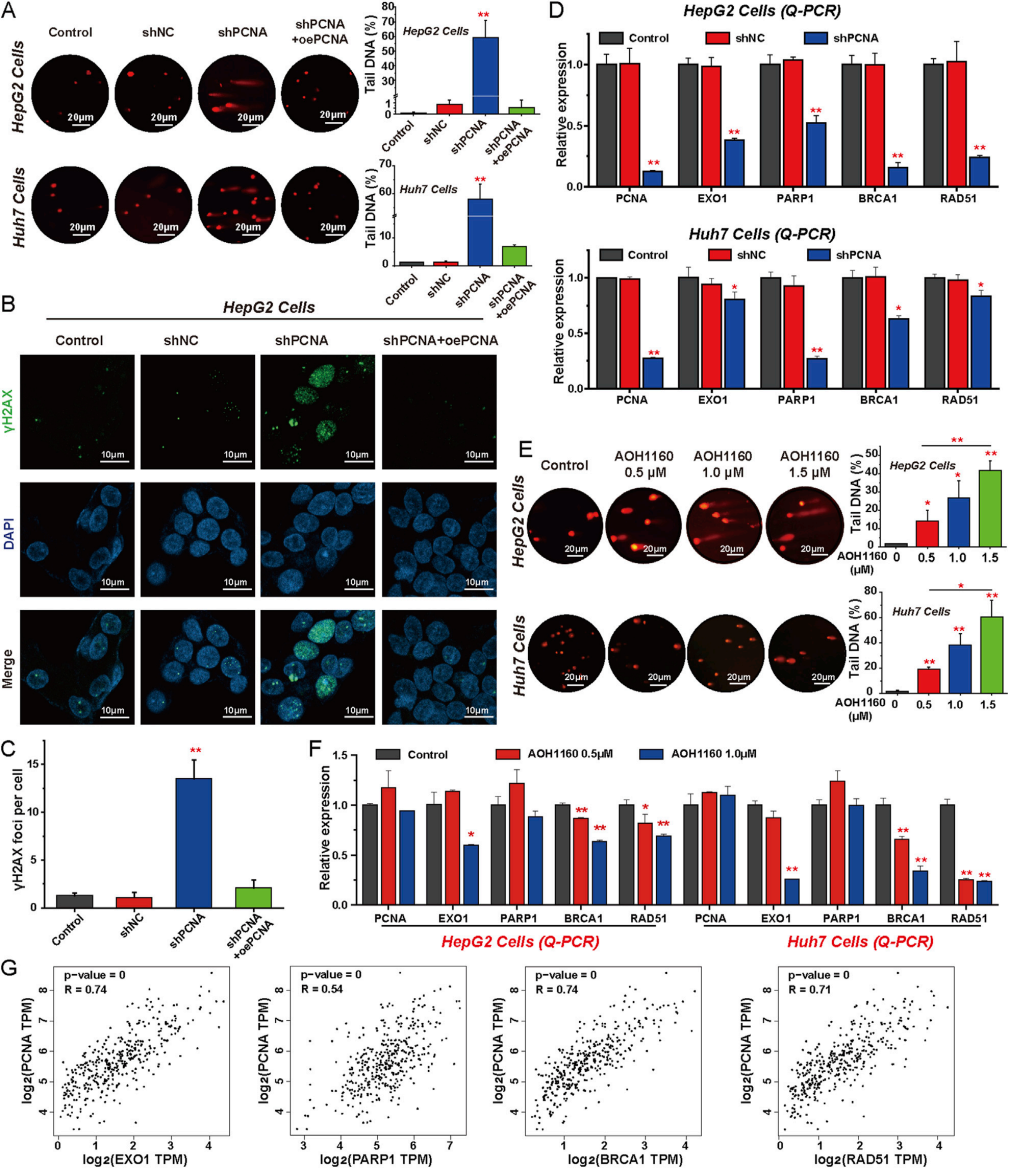
Repression of PCNA inhibits DNA repair in HCC cells. **(A)** The alkaline comet assay was performed to evaluate DNA damage in PCNA-knockdown HepG2 and Huh7 cells, with DNA damage levels quantified by measuring the percentage of tail DNA. **(B)** The foci of γH2AX were measured via immunofluorescence to evaluate the DNA double-strand break of HepG2 cells after PCNA knockdown. Magnification is ×100, scale bar = 10 μm. **(C)** Quantification of the number of γ-H2AX-positive foci in each cell based on immunofluorescence in HepG2 cells. **(D)** The Relative mRNA levels of indicated regulators of DNA repair following PCNA knockdown in HepG2 cells and Huh7 cells. **(E)** The effects of AOH1160 on DNA damage were assessed using the alkaline comet assay, with results quantified by measuring the percentage of tail DNA. **(F)** The effects of AOH1160 on the expression of DNA repair-related genes were analyzed by Q-PCR. **(G)** Expression correlation analysis of PCNA and key factors involved in DNA damage repair using the data from TCGA project. The results from three independent experiments were statistically analyzed using one-way ANOVA: *P < 0.05, **P < 0.01 compared with the control.

We next investigated the impact of PCNA depletion in the DNA damage repair of HCC cells. Damaged DNA has a tail in the comet assay that resembles a comet. Knockdown of PCNA significantly increased the number and extent of tailed DNA in HepG2 and Huh7 cells, indicating that repression of PCNA promoted DNA damage (Figure 3A). The accumulation of γH2AX foci also reflects the extent of DNA damage (Prabhu et al., 2024). Similarly, we found that the increased nuclear γH2AX foci was induced by PCNA silencing which was confirmed by confocal microscopy. However, overexpression of PCNA impaired the extent of DNA damage induced by knockdown of PCNA (Figures 3B, C). We then applied Q-PCR assays to assess the expression of genes-involved in DNA repair, including PARP1, EXO1, BRCA1, RAD51, X-ray repair cross complementing 1 (XRCC1), X-ray repair cross complementing 2 (XRCC2), breast cancer susceptibility gene 2 (BRCA2), partner and localizer of breast cancer 2 (PALB2), and DNA polymerase θ (POLQ), the mRNA levels of these genes were reduced in PCNA knockdown cells (Figure 3D; Supplementary Figure 1A). PCNA inhibitor AOH1160 also resulted in a dose-dependent increase in DNA damage in both HepG2 and Huh7 cells, as evidenced by the comet assay (Figure 3E). Correspondingly, Q-PCR analysis showed that AOH1160 significantly inhibited the expression of DNA repair genes in a dose-dependent manner (Figure 3F; Supplementary Figure 1B). Analysis of TCGA data demonstrated a significant positive correlation between PCNA expression and DNA damage repair-related gene expression in HCC (Figure 3F; Supplementary Figure 1C). These findings demonstrated that targeting PCNA genetically or pharmacologically inhibits DNA repair of HCC cells.

### 3.4 Inhibition of PCNA arrests cell cycle progression in HCC cells

**FIGURE 4:**
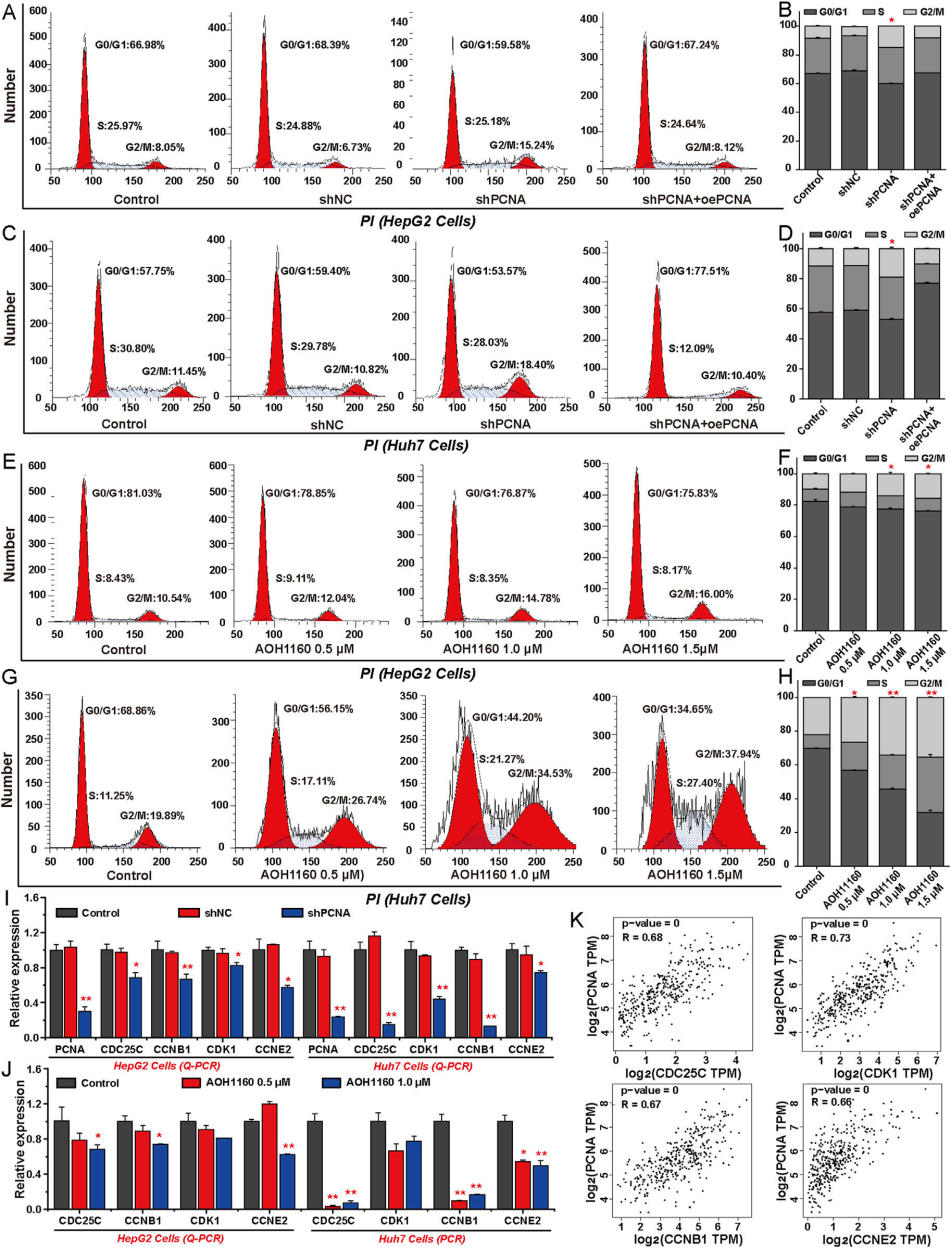
Inhibition of PCNA arrests cell cycle progression in HCC cells. **(A–D)** Cell cycle analysis was performed by flow cytometry in HepG2 cells and Huh7 cells with or without PCNA knockdown. **(E–H)** Cell cycle analysis was performed by flow cytometry in HepG2 cells and Huh7 cells treated with AOH1160. **(I)** Relative mRNA levels of indicated regulators of cell cycle progression following PCNA knockdown in HepG2 cells and Huh7 cells. **(J)** The effects of AOH1160 on the expression of genes involved in the cell cycle were analyzed by Q-PCR. **(K)** Expression correlation analysis of PCNA and the genes involved in cell cycle progression using the data from TCGA project. The results from three independent experiments were statistically analyzed using one-way ANOVA: *P < 0.05, **P < 0.01 compared with the control.

To assess the effect of targeting PCNA on cell cycle progression, flow cytometry analysis was performed in HCC cells. The analysis revealed that PCNA silencing increased the proportion of HepG2 and Huh7 cells in the G2/M phase, which may further lead to the death of HCC cells. In contrast, overexpression of PCNA reduced the proportion of the G2/M phase to accelerate the rate of cell mitosis (Figures 4A–D). Furthermore, AOH1160 inhibited the G2/M transition and arrested the cell cycle at the G2/M phase (Figures 4E–H). To further clarify the potential mechanisms, we evaluated the expression of genes-involved in cell cycle regulation using Q-PCR. The results demonstrated that inhibition of the expression or activity of PCNA decreased the expression levels of CDC25C, CCNB1, CDK1, CCNE2, cell-division cycle 25A (CDC25A), and cyclin-dependent kinase 2 (CDK2) (Figures 4I, J; Supplementary Figure 2A). Based on the data from TCGA project, correlation analysis revealed that PCNA expression was positively associated with the expression of CDC25C (R = 0.68), CDK1 (R = 0.73), CCNB1 (R = 0.67), CCNE2 (R = 0.66), CDC25A (R = 0.62), and CDK2 (R = 0.75) in HCC tissues (Figure 4K; Supplementary Figure 2B). These data suggested that inhibition of PCNA induced cell cycle arrest at G2/M phase in HCC cells.

### 3.5 PCNA directly interacted with PARP1 to promote HCC proliferation

**FIGURE 5:**
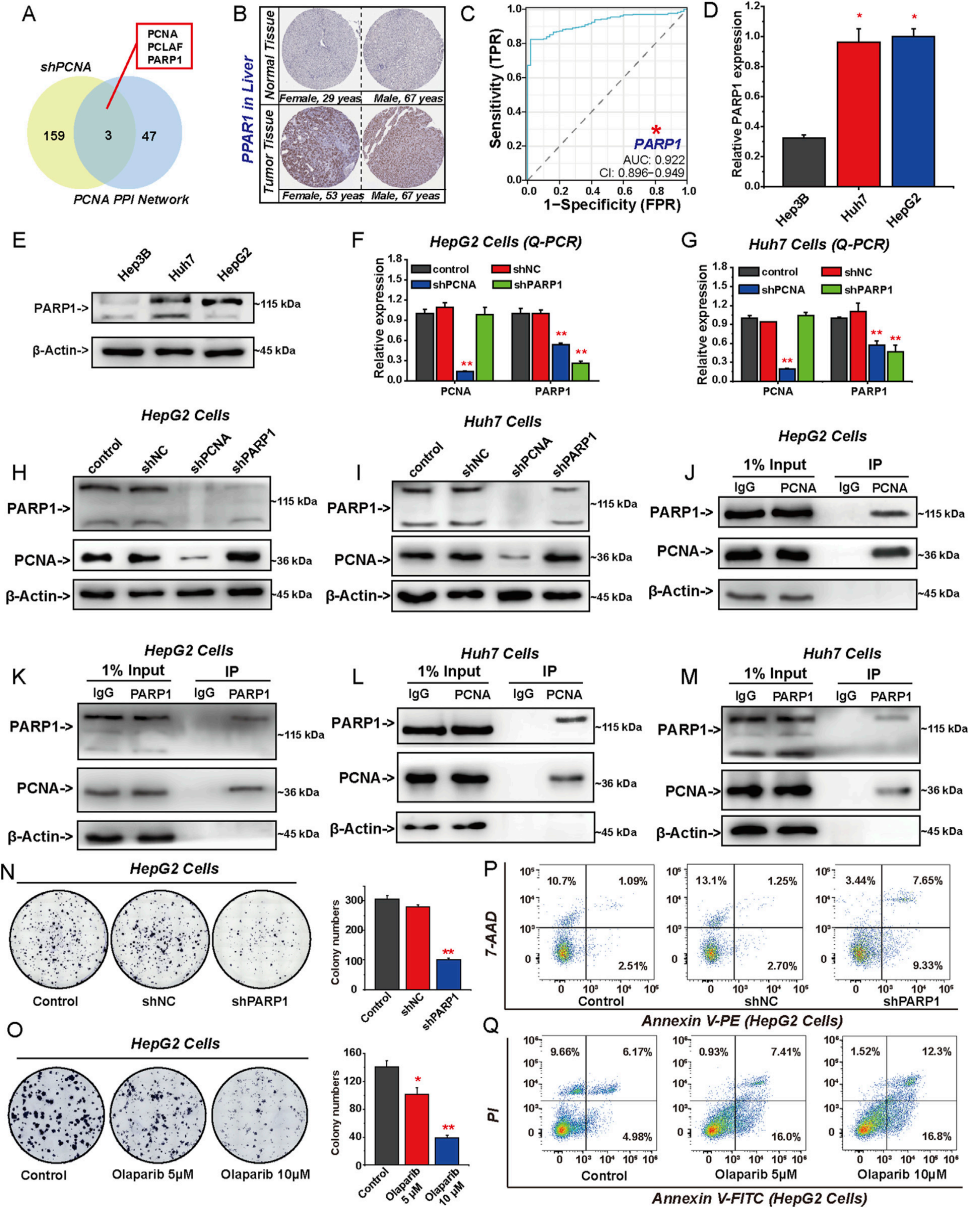
PCNA directly interacted with PARP1 to promote HCC proliferation. **(A)** Venn diagram for downregulated gene signature resulting from knockdown of PCNA and the PCNA PPI network. **(B)** Representative IHC staining intensity of PARP1 in HCC and normal tissues from the HPA databases. **(C)** ROC curves of PARP1 for HCC prediction in TCGA. The expression of PARP1 among different HCC cell lines was detected by Q-PCR **(D)** and Western blotting **(E)**. The expression of PCNA and PARP1 in HepG2 cells and Huh7 cells transduced with shPCNA or shPARP1 was analyzed by Q-PCR **(F, G)** and Western blotting **(H, I)**. **(J–M)** The relationship between PCNA and PARP1 was analyzed by Co-IP assay. Colony formation assays were performed to analyze proliferation in PARP1-knockdown **(N)** and Olaparib-treated **(O)** HepG2 cells, followed by quantification of colony numbers. Apoptosis of HepG2 cells with PARP1 knockdown **(P)** or Olaparib **(Q)** was assessed by flow cytometry. The results from three independent experiments were statistically analyzed using one-way ANOVA: *P < 0.05, **P < 0.01.

To delineate the molecular mechanism of PCNA in HCC tumorigenesis, we systematically identified key effector proteins downstream of PCNA. Through Venn diagram analysis integrating DEGs downregulated upon PCNA silencing with genes within the PCNA protein-protein interaction (PPI) network, we identified three overlapping candidates: PCNA, PCLAF, and PARP1. Structural and functional analyses confirmed that PCNA interacts with PCLAF to orchestrate its canonical roles in DNA replication and repair. Building on this foundation, we further discovered that PCNA mechanistically modulates PARP1 expression (Figure 5A). Based on these findings, we postulated that PARP1 may serve as the pivotal mechanistic target through which PCNA drives HCC pathogenesis. Subsequently, we explored that the expression of PARP1 is significantly higher in HCC tissues compared to normal adjacent tissues (Figure 5B). The ROC curve analysis indicates that the AUC value of PARP1 is 0.922, suggesting a high diagnostic value (Figure 5C). Correlation analysis also showed that PARP1 was associated with DNA repair-related genes and cell cycle regulation genes (Supplementary Figure 3). And we also that the differential expression of PARP1 among different HCC cell lines was similar to PCNA, which was highest in HepG2 cells (Figures 5D, E). Surprisingly, knockdown of PCNA inhibited the expression of PARP1 in mRNA and protein levels, however, knockdown of PARP1 had no effect on the expression of PCNA (Figures 5F–I). Indeed, Co-IP assay suggested that PCNA directly interacted with PARP1 (Figure 5J-M). Collectively, these results indicate that PARP1 is the downstream target of PCNA and directly interacts with PCNA. We further explored the effect of PARP1 on the proliferation of HepG2 cells. Inhibition expression or enzymatic activity of PARP1 significantly inhibited the formation of HepG2 clones (Figures 5N, O), and promoted the apoptosis of HepG2 cells (Figures 5P, Q). In summary, these results suggest that PARP1 is the key downstream target of PCNA-mediated HCC proliferation.

### 3.6 Knockdown of PCNA increases the sensitivity of HCC cells to PARP1 inhibitor olaparib

**FIGURE 6:**
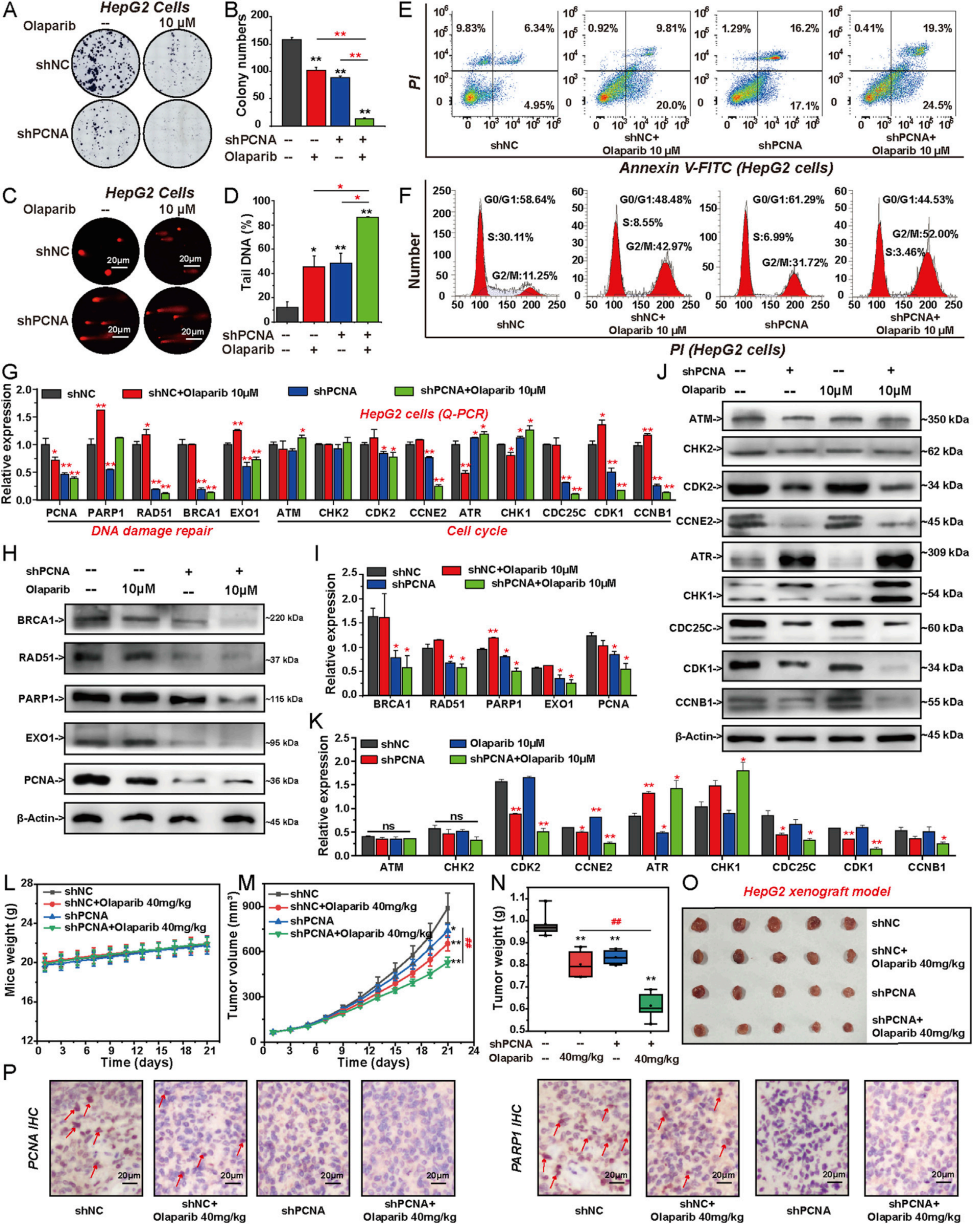
Knockdown of PCNA increases the sensitivity of HCC cells to PARP1 inhibitor Olaparib. HepG2 cells were transfected with shNC or shPCNA and followed by Olaparib (10 μM) treatment for 6 days. **(A, B)** Proliferation was detected by colony formation assay, followed by quantification of colony numbers. **(C, D)** DNA damage level was assessed by alkaline comet assay, followed by quantification of the percentage of tail DNA. **(E)** Apoptosis was assessed by flow cytometry with PI and annexin V-FITC double staining. **(F)** Cell cycle analyses were conducted by flow cytometry. **(G)** Relative expression of DNA repair-related genes and factors involved in cell cycle progression were analyzed by Q-PCR. **(H, I)** The protein expression levels of DNA repair-related factors following each individual treatment were analyzed via Western blotting. **(J, K)** The protein expression levels of factors involved in cell cycle progression were analyzed by Western blotting. The effects of shPCNA, Olaparib, and their combination on mice weight **(L)**, tumor volume **(M)**, and tumor weight **(N)**. The photos of tumor nodules **(O)** in each group. **(P)** Immunohistochemical staining of PCNA and PARP1 in the isolated xenograft tumor. The results from three independent experiments were statistically analyzed using one-way ANOVA: *P < 0.05, **P < 0.01 compared with the shNC group; #P < 0.05, ##P < 0.01 compared with the shPCNA/Olaparib combined group (Olaparib: 40 mg/kg).

To investigate the effect of knocking down PCNA on the sensitivity of HCC to PARP1 inhibitor, HepG2 cells were treated with shPCNA or 10 µM Olaparib for 6 days separately or in combination. Both Olaparib and shPCNA inhibited the proliferation and DNA repair of HepG2 cells and promoted cell apoptosis (Figures 6A–E). Besides, Olaparib and shPCNA inhibited the cell cycle transition from G2 phase to M phase in HepG2 cells (Figure 6F). Compared with the shPCNA infected group and Olaparib treated group, the combination of shPCNA and Olaparib significantly inhibited the proliferation and DNA repair of HepG2 cells, promoted cancer cell apoptosis, and arrested the cell cycle at G2/M phase (Figures 6A–F). Q-PCR and Western blotting were used to investigate the mRNA and protein levels of factors-involved in DNA repair and cell cycle progression (Figures 6G–K). Knockdown of PCNA significantly inhibited the expression of PARP1, RAD51, BRCA1, and EXO1 in both mRNA and protein levels. Olaparib significantly promoted the expression of PARP1, RAD51 and EXO1 after 6 days of treatment. The combination of shPCNA and Olaparib induced a significant decrease of these DNA repair-related genes (Figures 6G–I). Subsequently, we investigated the effects of shPCNA, Olaparib, and their combination on cell cycle regulatory genes. Knockdown of PCNA significantly promoted the expression of ATR and CHK1, and inhibited the expression of CDC25C, CDK1, CCNB1, CDK2 and CCNE2, but did not affect the expression of ATM and CHK2. Olaparib promoted the expression of CDK1, CCNB1 and CCNE2. The combination group significantly promoted the expression of ATR and CHK1, and inhibited the expression of CDC25C, CDK1, CCNB1, CDK2, and CCNE2 in mRNA and protein levels (Figures 6G, J, K). We also investigated the effect of knocking down PCNA on the sensitivity to PARP1 inhibitor Olaparib *in vivo*. To construct the xenograft tumor mouse model, PCNA-knockdown stable HepG2 cell lines were subcutaneously injected into nude mice. Subsequently, Olaparib treatment was performed once a day at 40 mg/kg for 21 days, and the tumor growth curve was detected. As shown in Figure 6L-O, knockdown of PCNA or treatment with Olaparib significantly inhibited the growth of HepG2 xenograft tumors. Compared with the shPCNA infection group and Olaparib treatment group, the combined treatment group had a more significant inhibition effect on HepG2 xenograft tumor growth. Further research showed that knockdown of PCNA significantly repressed the expression of PCNA and PARP1 in HepG2 xenograft tumor tissues. Olaparib decreased PCNA expression and increased PARP1 expression. Their combination significantly inhibited the expression of PCNA and PARP1 (Figure 6P). Collectively, these results suggest that knockdown of PCNA promotes the sensitivity of HCC cells to Olaparib *in vitro* and *in vivo*.

### 3.7 Combined inhibition of PCNA and PARP1 has a synergistic effect on HCC cells

**FIGURE 7:**
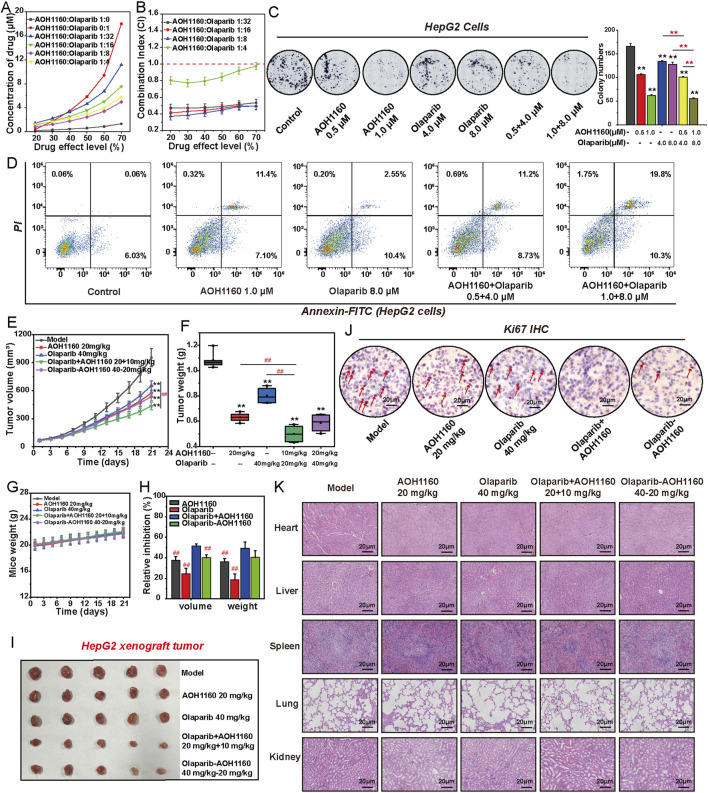
Combined inhibition of PCNA and PARP1 has a synergistic effect on HCC cells. **(A)** The effects of combining AOH1160 and Olaparib on the proliferation of HepG2 cells. **(B)** CI values for concurrent treatment with AOH1160 and Olaparib in HepG2 cells. **(C)** The effects of AOH1160 and/or Olaparib on the proliferation of HepG2 cells were measured by colony formation assay, followed by quantification of colony numbers. **(D)** The effects of AOH1160 and/or Olaparib on the apoptosis of HepG2 cells. **(E–I)** HepG2 cells were injected into nude mice and the mice were subsequently treated with AOH1160 and/or Olaparib at the indicated times. The tumor volume **(E)**, tumor weight **(F)**, mice weight **(G)**, relative volume and weight inhibition **(H)**, and tumor nodules **(I)** in each group. **(J)** Immunohistochemical staining of Ki67 in the isolated xenograft tumor. **(K)** Tissue damage was determined by H&E staining. The results from three independent experiments were statistically analyzed using one-way ANOVA: *P < 0.05, **P < 0.01 compared with the control; #P < 0.05, ##P < 0.01 compared with the AOH1160/Olaparib combined group (AOH1160/Olaparib: 10 + 20 mg/kg).

To evaluate the therapeutic potentials of targeting PCNA and PAPR1, we treated HepG2 cells with AOH1160/Olaparib alone or in combination for 6 days to assess their anticancer efficiency. The effect of AOH1160 and Olaparib (1:32, 1:16, 1:8, 1:4) in combination were examined in HepG2 cells (Figure 7A). The drug combination indexes (CI) values were less than 1.0 for all concentration groups, suggesting a synergistic effect between AOH1160 and PARP1 at the indicated concentrations (Figure 7B). The dose ratio of 1:8 induced the most significant inhibition (CI < 0.5), therefore, we selected the dose ratio of 1:8 for further study. Compared with AOH1160 (1.0 μM) alone- and Olaparib (8.0 μM) alone-treated group, the combination of AOH1160 (0.5 μM) and Olaparib (4.0 μM) at lower concentrations could significantly inhibit the clonogenic growth of HepG2 cells (Figure 7C). Meanwhile, AOH1160 and Olaparib synergistically promoted the apoptosis of HepG2 cells after 6 days of treatment (Figure 7D). To further evaluate the anticancer effect of the combined inhibition of PCNA and PARP1 *in vivo*, BALB/c nude mice were subcutaneously injected with HepG2 cells. Approximately 1 week later, the mice were equally divided into five groups and intraperitoneally injected with 20 mg/kg AOH1160, 40 mg/kg Olaparib, a combination of both drugs (10 mg/kg AOH1160 plus 20 mg/kg Olaparib), a sequential treatment (40 mg/kg Olaparib for 10 days, followed by 20 mg/kg AOH1160 for 10 days), or an equal volume of saline as a control group. After 21 days of treatment, AOH1160 and Olaparib synergistically inhibited tumor size and tumor weight but did not affect the body weight of mice at our tested dosages, and it also reduced the expression of Ki67 (Figures 7E–J). After analysis of H&E-stained organs, we found that AOH1160, Olaparib, and their combination did not cause significant damage to the kidney, lung, spleen, liver, or heart (Figure 7K). Together, these results suggested that AOH1160 and Olaparib synergistically inhibited the malignant proliferation of HepG2 cells *in vitro* and *in vivo*.

### 3.8 AOH1160 and olaparib synergistically inhibit DNA damage repair and cell cycle progression

**FIGURE 8:**
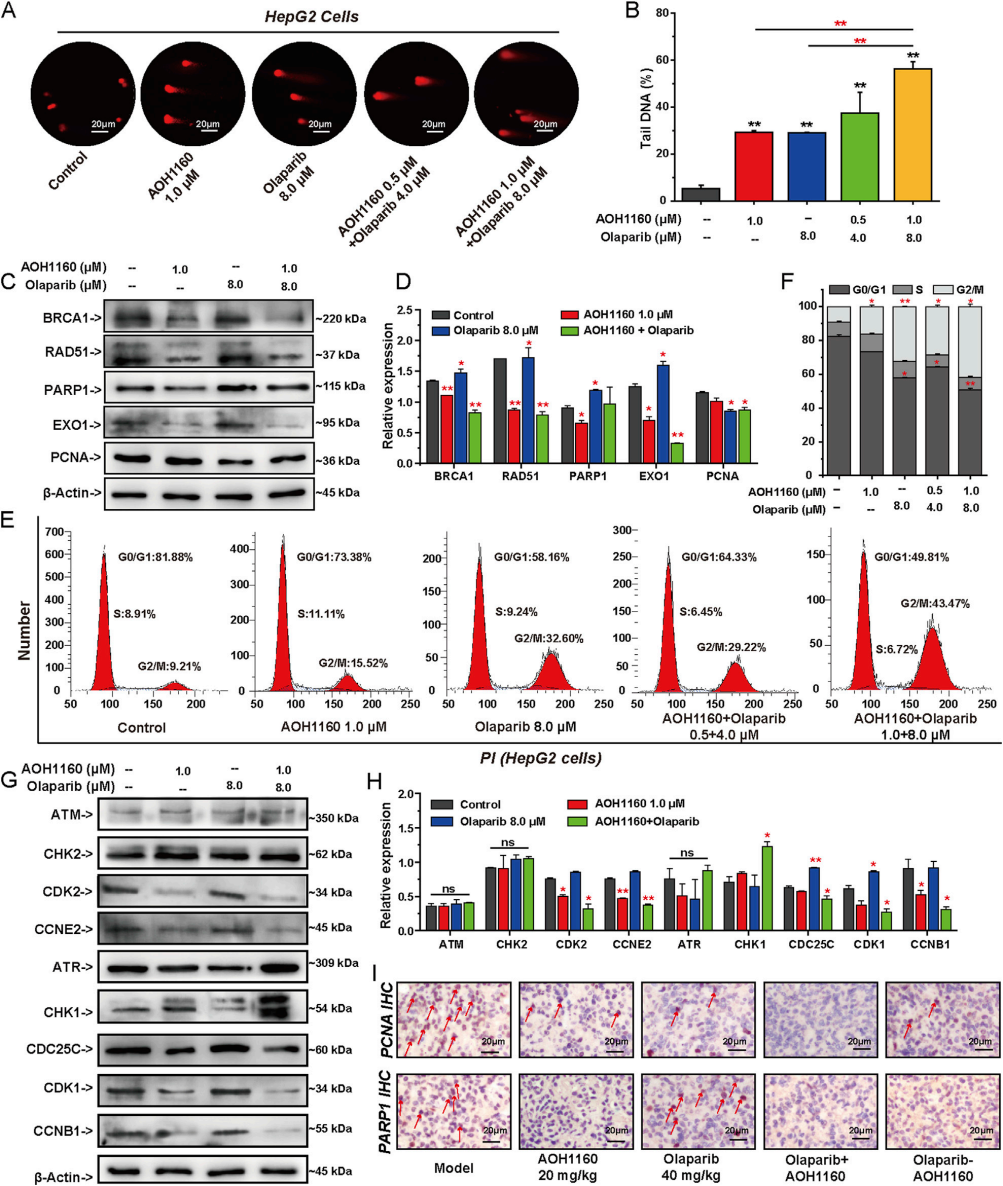
AOH1160 and Olaparib synergistically inhibit DNA damage repair and cell cycle progression. **(A)** The effects of AOH1160 and/or Olaparib on DNA damage detected by alkaline comet assay. **(B)** Quantified results by the percentage of tail DNA in the comet assay. **(C, D)** The effects of AOH1160 and/or Olaparib on the expression of DNA repair-related proteins were analyzed by Western blotting in HepG2 cells. **(E, F)** The effects of AOH1160 and/or Olaparib on cell cycle progression in HepG2 cells. **(G, H)** The effects of AOH1160 and/or Olaparib on the expression of proteins involved in cell cycle control were analyzed by Western blotting in HepG2 cells. **(I)** Effects of AOH1160 and/or Olaparib on the expression of PCNA and PARP1 *in vivo* analyzed by immunohistochemistry.

To evaluate the effect of AOH1160 and Olaparib in combination on DNA repair and cell cycle progression, we conducted a series of *in vitro* experiments (Figures 8A–H). Compared with single treatment, DNA damage was more pronounced in the AOH1160 and Olaparib combined group, as determined by the comet assay (Figures 8A, B). Western blots were used to analyze the expression of proteins involved in DNA repair, Olaparib significantly promoted the expression of PARP1, RAD51 and EXO1, AOH1160 significantly inhibited the expression of PARP1, BRCA1, EXO1, and RAD51, but did not affect the expression of PCNA. The combination of AOH1160 and Olaparib synergistically inhibited the expression of BRCA1, EXO1, XRCC1, XRCC2, and RAD51, but did not affect the expression of PARP1 and PCNA (Figures 8C, D). AOH1160 (1.0 μM) inhibited the G2/M transition and arrested the cell cycle at the G2/M phase. Olaparib (8.0 μM) promoted the G1/S transition and arrested the cell cycle at the G2/M phases. Combining AOH1160 (1.0 μM) with Olaparib (8.0 μM) arrested the cell cycle at the G2/M phase (Figures 8E,F). Western blots were used to analyze the expression of proteins involved in cell cycle progression (Figures 8G, H). Olaparib significantly promoted the expression of CDC25C, and CDK1 to accelerate mitosis. AOH1160 significantly inhibited the expression of CCNB1, CDK2 and CCNE2. The combination of AOH1160 and Olaparib promoted the expression of ATR and CHK1, and inhibited the expression of CDC25C, CDK1, CCNB1, CDK2 and CCNE2. AOH1160 impaired Olaparib-induced expression of these proteins in HepG2 cells. IHC staining for PCNA and PARP1 confirmed that Olaparib combined with AOH1160 could induce the down expression of PARP1 and PCNA respectively (Figure 8I). Overall, these findings indicated that PCNA and PARP1 depletion synergistically inhibit DNA repair and cell cycle progression.

## 4 Discussion

HCC is the leading causes of cancer-related death worldwide, due to mild symptoms at early stage and almost half of the patients are diagnosed at advanced stage (Sadagopan and He, 2024; Wang et al., 2024). With advances in sequencing technology and bioinformatics, innovative targeted therapies can potentially improve survival and quality of life for patients with challenging HCC (Wang et al., 2024; Becht et al., 2024). The success of antiangiogenic agents and tyrosine kinase inhibitors in treating HCC demonstrates that targeted therapy is another option and hope for the treatment of challenging HCC (Becht et al., 2024). However, existing targeted drugs have limitations such as drug resistance, significant side effects, narrow clinical indications, and high tendency for relapse (Lei et al., 2024). Therefore, novel targets and strategies are urgently required for the treatment of HCC. Previous studies have found that PCNA is highly expressed in various types of tumors and contributes to malignant progression and poor prognosis (Lv et al., 2016; Gu et al., 2023). Targeting PCNA in monotherapy or combination therapy may have a good therapeutic effect on HCC (Cheng et al., 2020). Herein, we investigated the mechanism of PCNA in regulating the malignant progression of HCC cells from the perspective of regulating DNA repair and cell cycle progression.

PCNA is the center of DNA replication and DNA damage repair that plays an important role in maintaining genomic integrity and preventing the propagation of DNA errors (González-Magaña and Blanco, 2020). PCNA is highly expressed in many cancers and contributes to malignant proliferation and poor prognosis (Lv et al., 2016). Consistently, our research confirmed that PCNA is overexpressed in HCC cells and high expression of PCNA is associated with poor prognosis and short survival in HCC patients. Additionally, we found that suppression of PCNA with shPCNA or AOH1160 significantly inhibited the proliferation of HCC cells both *in vitro* and *in vivo*. AOH1160 is a PCNA inhibitor that can selectively kill many types of cancer cells at below micromolar concentrations through specifically targeting the L126-Y133 region of PCNA in cancer cells, without causing significant toxicity to a broad range of nonmalignant cells (Gu et al., 2018). To elucidate the specific mechanisms, we performed RNA sequencing to analyze the effect of PCNA on total mRNA expression in HepG2 cells. The result showed that PCNA promoted the malignant progression of HCC cells by regulating cell cycle progression and DNA damage repair. Further mechanism studies showed that knockdown of PCNA inhibited the expression of EXO1, BRCA1, RAD51, PARP1, and other genes-involved in DNA damage repair. The long-range end-resection factor EXO1 is a multipotent DNA exonerase that is mainly involved in HR repair and non-homologous end-link repair (Gioia et al., 2023; van de Kooij et al., 2024). BRCA1 and RAD51 are the essential factors involved in HR repair pathway that trigger the precise repair of DNA DSBs (Zhao et al., 2017). In cells lacking EXO1, BRCA1 and RAD51, the precise DSB repair pathway does not work properly (van de Kooij et al., 2024; Zhao et al., 2017). PARP1 is involved in DNA SSBs repair through the BER pathway. The effect of PCNA on the expression of these genes suggested that PCNA could repair both SSBs and DSBs of DNA through multiple pathways. Precise DNA damage repair occurs in the S and G2 phases of the cell cycle, especially in the HR repair pathway (Bournaka et al., 2024). Indeed, knockdown of PCNA blocked the cell cycle in the G2/M phase and inhibited the expression of CDC25C, CDK1, and CCNB1. CDC25C is a cell cycle regulatory protein that can activate CDK1 and CCNB1 to promote cell mitosis (Liu et al., 2020). The combination of CDK1 and CCNB1 can promote cells from G2 phase to M phase and ensure normal cell division and proliferation (Smith et al., 2020). Decreased expression of CDC25C, CDK1, and CCNB1 inhibited the transfer of cell cycle from G2 phase to M phase, resulting in the inhibition of cancer cell proliferation (Liu et al., 2020; Smith et al., 2020). When the cell cycle is blocked in the G2 phase, precise DNA repair is initiated. If DNA cannot be repaired in this phase, large amounts of DNA fragments accumulate, leading to chromosomal instability and cell death (Wang et al., 2021; Smith et al., 2020). The regulatory effects of PCNA on DNA repair and cell cycle progression suggest that PCNA is a key driving target for HCC progression and poor prognosis. Targeting PCNA has the potential to improve survival and quality of life for patients with advanced and metastasis HCC.

Subsequently, we identified PARP1 as the key target involved in the PCNA-mediated HCC proliferation. While previous studies have established that PCNA orchestrates DNA replication and repair via its functional interplay with PCLAF (De March et al., 2018), our work extends this paradigm by revealing the role of PCNA as a modulator of PARP1 expression. To dissect the cooperative mechanism underlying PCNA-PARP1 oncogenic activity, we employed Co-IP assays and confirmed their direct interaction. Our results position PARP1 as a downstream effector of PCNA and suggest its potential integration into a tripartite regulatory complex involving PCNA and PCLAF. This cooperative interaction likely fine-tunes DNA replication fidelity and repair pathway selection. Clinically, PARP inhibitors such as Olaparib have demonstrated synthetic lethality in homologous recombination (HR)-deficient cancers. Emerging strategies aim to overcome PARP inhibitors resistance and broaden their utility to HR-proficient malignancies, including HCC. Based on our findings, we propose that PCNA inhibition may sensitize HR-competent HCC to PARP inhibitors by destabilizing the PCNA/PARP1 axis, thereby exacerbating replication stress-induced genomic instability. This synergistic therapeutic vulnerability could expand PARP inhibitors applications in HCC treatment, particularly in tumors resistant to conventional therapies. Supporting this hypothesis, we used shPCNA or AOH1160 to inhibit the expression or activity of PCNA and investigated the regulatory effect of PCNA on the sensitivity of HepG2 cells to Olaparib *in vitro* and *in vivo*. Knockdown of PCNA promoted the inhibitory effect of Olaparib on the malignant proliferation of HepG2 cells *in vitro* and *in vivo*. Meanwhile, silencing PCNA expression enhanced the effect of Olaparib on cell apoptosis and DNA repair and cell cycle progression. Moreover, AOH1160 and Olaparib synergistically inhibited the proliferation of HepG2 cells and HepG2 xenograft tumors. Further mechanistic studies showed that knockdown of PCNA inhibited the expression of BRCA1, RAD51, XRCC1, and EXO1. AOH1160 also inhibited the expression of these DNA repair-related genes. The reduction of these genes would block HR-mediated repair, thereby increasing the sensitivity of HCC cells to Olaparib. Olaparib induced the upregulation of PARP1 and RAD51, a compensatory feedback mechanism activated in response to persistent DNA damage, which may drive the development of therapeutic resistance (Fu et al., 2024; Liu et al., 2024). Suppression of PCNA impaired Olaparib-induced overexpression of PARP1 and RAD51, thereby reversing the resistance of cancer cells to Olaparib. Meanwhile, we found that inhibition of PCNA with AOH1160 and inhibition of PARP1 with Olaparib could arrest the cell cycle at the G2/M phases. Olaparib significantly promoted the expression of CCNE2 to accelerate mitosis (Smith et al., 2020; Ditano et al., 2021). Olaparib-induced accelerating of cell cycle progression was beneficial for HR-mediated DNA repair (Wicks et al., 2022). AOH1160 repressed the expression of CDK1, CCNB1, CDK2 and CCNE2 to arrest the cell cycle at G2/M phase, thus blocking the promotion of cancer cell mitosis. The combination of Olaparib and AOH1160 promoted the expression of ATR and CHK1, inhibited the expression of CD25C, CDK1, CCNB1, CDK2 and CCNE2. DNA single-strand breaks activate ATR, which phosphorylates and activates CHK1. Activated CHK1 then phosphorylates CDC25C, resulting in its inactivation and maintaining CDK1 in an inactive phosphorylated state, thereby arrested the cell cycle at G2/M phase to inhibit the malignant proliferation of HCC cells (Smith et al., 2020). However, neither AOH1160 nor Olaparib affects the expression of ATM and CHK2, whether they influence the phosphorylation of these targets will be explored in our further studies.

In conclusion, we clarified the mechanism for the effect of PCNA on the proliferation of challenging HCC *in vitro* and *in vivo*. PCNA is overexpressed in HCC cells and closely correlated with the poor prognosis of HCC patients. PCNA promotes the proliferation and progression of HCC by regulating DNA repair and cell cycle progression. Mechanistically, PARP1 is the downstream target of PCNA and directly interacts with PCNA. Targeting PCNA increase the sensitivity of HCC cells to Olaparib. Further AOH1160 and Olaparib synergistically inhibited the proliferation, DNA damage repair and cell cycle progression of HCC cells. The present findings support the premise coinhibition of PCNA and PAPR1 for the treatment of advanced HCC. Collectively, the study provides a mechanistic foundation for therapies targeting PCNA/PARP1 axis and the development of dual-target PCNA/PARP1 inhibitors.

## Data Availability

The original contributions presented in the study are publicly available. This data can be found here: https://www.ncbi.nlm.nih.gov/, accession number PRJNA1247785.
