# Supplementary material for: Targeting PCNA/PARP1 axis inhibits the malignant progression of hepatocellular carcinoma
Source: Front Pharmacol. 2025 Apr 17;16:1571786. doi: 10.3389/fphar.2025.1571786 (PMC12043649; doi:10.3389/fphar.2025.1571786)
Supplement: Supplementary file 1 [file DataSheet1.docx]

**Supplemental data**

for

**Targeting PCNA/PARP1 axis inhibits the malignant progression of hepatocellular carcinoma**

**Running title:** Targeting PCNA/PARP1 axis for hepatocellular carcinoma

Jipin Li^1,2^^†^, Tao Yong^1,2†^, Yali Chen^2^, Tingyu Zeng^2^, Kaifeng Zhang^2^, Shuping Wang^2*^, Youcheng Zhang^1*^

^1^Department of General Surgery, The Second Hospital of Lanzhou University, Lanzhou 730030, China

^2^Key Laboratory of Preclinical Study for New Drugs of Gansu Province, Institute of Biochemistry and Molecular Biology, School of Basic Medical Sciences, Lanzhou University, Lanzhou 730000, China

^†^These authors have contributed equally to this work.

*Correspondence: Youcheng Zhang, e-mail: zhangyouchengphd@163.com; Shuping Wang, e-mail: wangsp16@126.com

1. **Materials and methods**
   1. **Construction and confirmation of human PCNA lentiviral recombination vector**

Lentiviral recombination vector of human PCNA genes was constructed and purchased from Genechem Co. Ltd. (Shanghai, China). Lentiviral GV492 was used as vector to express human PCNA gene, and the restriction sites are BamHI/AgeI. The recombination vector of GV492-PCNA were confirmed by PCR and sequencing.

- 1. **Construction and confirmation of human shPCNA and shPARP1 lentiviral recombination vector**

Lentiviral recombination vector of the short-hairpin RNA against PCNA and the lentiviral recombination vector of the short-hairpin RNA against PARP1 were constructed and purchased from Genechem Co. Ltd. (Shanghai, China). Lentiviral GV248 was used as a vector to express PCNA shRNA and PARP1 shRNA. The restriction sites are BamHI and AgeI. The recombination vectors of pGV248-shPCNA and pGV248-shPARP1 were confirmed by sequencing and Q-PCR.

1. **Supplemental Tables**

**Supplemental Table 1.** List of antibodies.

| **Name** | **Manufacturer** | **Cat. no.** |
| --- | --- | --- |
| anti-PARP1 | Abcam (Cambridge, England) | ab227244 |
| anti-EXO1 | Abcam (Cambridge, England) | ab95068 |
| anti-CCNE2 | Abcam (Cambridge, England) | ab40890 |
| anti-CDK1 | Abcam (Cambridge, England) | ab133327 |
| anti-CCNB1 | Abcam (Cambridge, England) | ab32053 |
| anti-BRCA1 | Cell Signaling Technology (Boston, MA, USA) | 14823 |
| anti-RAD51 | Cell Signaling Technology (Boston, MA, USA) | 8875 |
| anti-CDC25C | Cell Signaling Technology (Boston, MA, USA) | 4688 |
| anti-CDK2 | Cell Signaling Technology (Boston, MA, USA) | 2546 |
| anti-γH2AX | Cell Signaling Technology (Boston, MA, USA) | 9718 |
| Anti-rabbit IgG (H + L), F(ab’)2 Fragment (Alexa Fluor® 488 Conjugate) | Cell Signaling Technology (Boston, MA, USA) | 4412 |
| anti-PCNA | Proteintech (Wuhan, China) | 10205-2-AP |
| anti-XRCC2 | Proteintech (Wuhan, China) | 66652-1-Ig |
| anti-XRCC1 | Proteintech (Wuhan, China) | 21468-1-AP |
| anti-β-actin | Proteintech (Wuhan, China) | 66009-1-Ig |
| HRP-Goat Anti-Mouse | Proteintech (Wuhan, China) | RGAM001 |
| HRP-Goat Anti-Rabbit | Proteintech (Wuhan, China) | RGAR001 |

**Supplemental Table 2.** Primers used in this study.

| **Name** | **Sense (5’-3’)** | **Antisense (5’-3’)** |
| --- | --- | --- |
| PCNA | ATTTGCACGTATATGCCGAGA | TGCAGAAAATTTCACTCCGTCT |
| PARP1 | ACACAATGCGTATGACTTGGA | CCGTGCCACAGCAATCTTCG |
| XRCC1 | CTCAAGGCAGACACTTACCGAA | AGCACCTCCACGAAAGCTGA |
| XRCC2 | CCTTTTGATTTTGGATAGCCT | GCGATAGTCATTTACAAGCTTC |
| RAD51 | CCCATTTCACGGTTAGAGCA | CTTTGGCTTCACTAATTCCCT |
| BRCA1 | TACTAGGCATAGCACCGTTG | ATGCCTTTGCCAATATTACCTG |
| BRCA2 | TGAAATTAAACGGAAGTTTGC | GAATAAAAGCCCCTAAACCC |
| PALB2 | TCCCAAAAGGCCAAACTCG | GTCATTATCATCAGGCGCAAC |
| POLQ | AATGGTCTGATCAATCGCCTC | ACAAGATGCTGATTTCCGAGT |
| EXO1 | GAAGGCAATAAATCATTGAGC | TGCAAATTTATTTCTCGTCCT |
| CDC25A | CCTCCGAGTCAACAGATTCAGG | CTTCAGAGCTGGACTACATCCC |
| CDK2 | GCTTTCTGCCATTCTCATCGG | TGGCTAGTCCAAAGTCTGC |
| CCNE2 | TCCAAGAGTTTGCTTACGTCA | TTTAGGAGCATCTTTAAGAGC |
| CDC25C | CACTTCCTTTACCGTCTGTCC | CTGAGTGGCAGTTATCTCCC |
| CDK1 | ATTTGGAGTATAGGCACCAT | GCCACACTTCATTATTGGGA |
| CCNB1 | TGAGAGCCATCCTAATTGACT | AATTATTCTGCATGAACCGAT |
| GAPDH | GAAACTGTGGCGTGATGGC | CACCACTGACACGTTGGCAG |

1. **Supplemental** **Figures**

**Supplemental Figure 1**


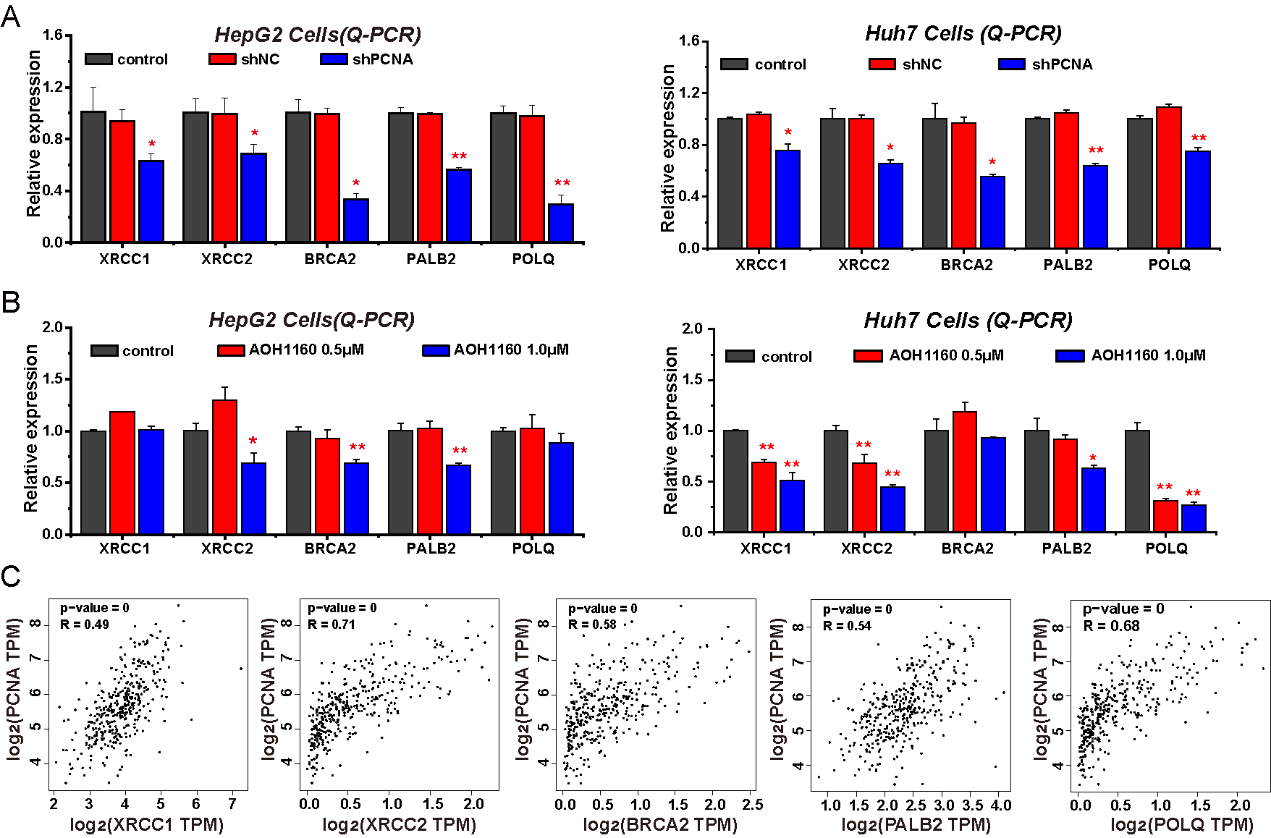


**Supplemental Figure 1. Repression of PCNA inhibits DNA repair in HCC cells.** (A) The effects of shPCNA on the expression of XRCC1, XRCC2, BRCA2, PALB2, and POLQ analyzed by Q-PCR in HepG2 and Huh7 cells. (B) The effects of AOH1160 on the expression of XRCC1, XRCC2, BRCA2, PALB2, and POLQ analyzed by Q-PCR in HepG2 and Huh7 cells. (C) Expression correlation analysis of PCNA with XRCC1, XRCC2, BRCA2, PALB2, and POLQ in HCC. The results from three independent experiments were statistically analyzed using one-way ANOVA: ^*^P<0.05, ^**^P<0.01 compared with the control.

**Supplemental Figure 2**


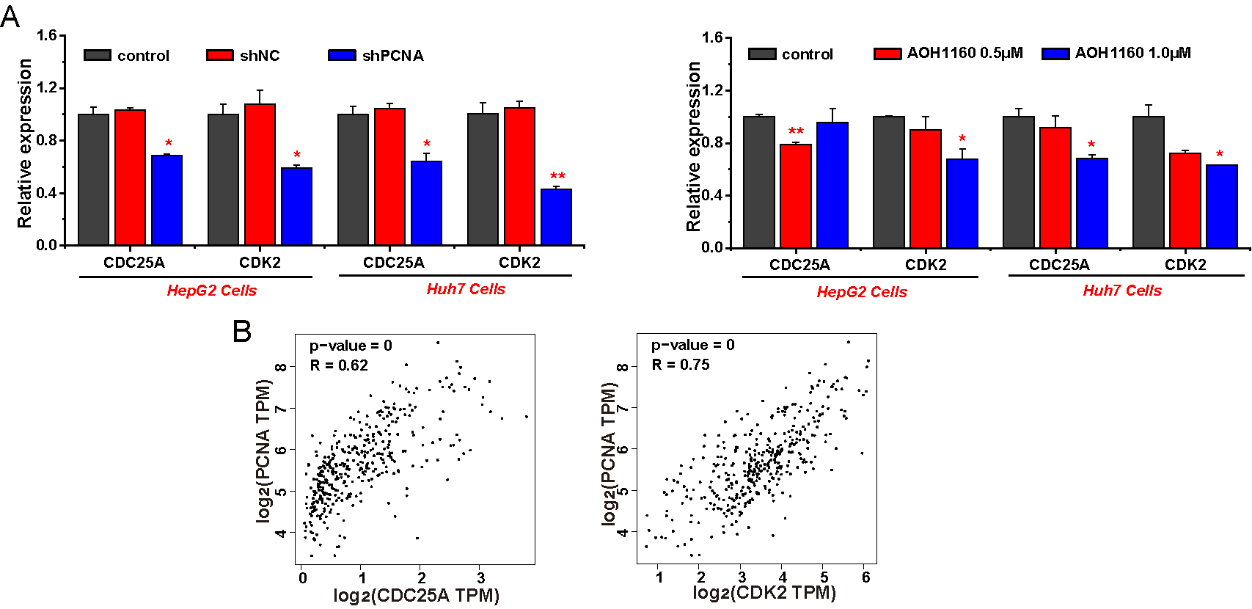


**Supplemental Figure 2. Inhibition of PCNA arrests cell cycle progression in HCC cells.** (A) The effects of shPCNA and AOH1160 on the expression of CDC25A and CDK2 analyzed by Q-PCR in HepG2 and Huh7 cells. The results from three independent experiments were statistically analyzed using one-way ANOVA: ^*^P<0.05, ^**^P<0.01 compared with the control. (B) Expression correlation analysis of PCNA with CDC25A and CDK2 in HCC.

**Supplemental Figure 3**


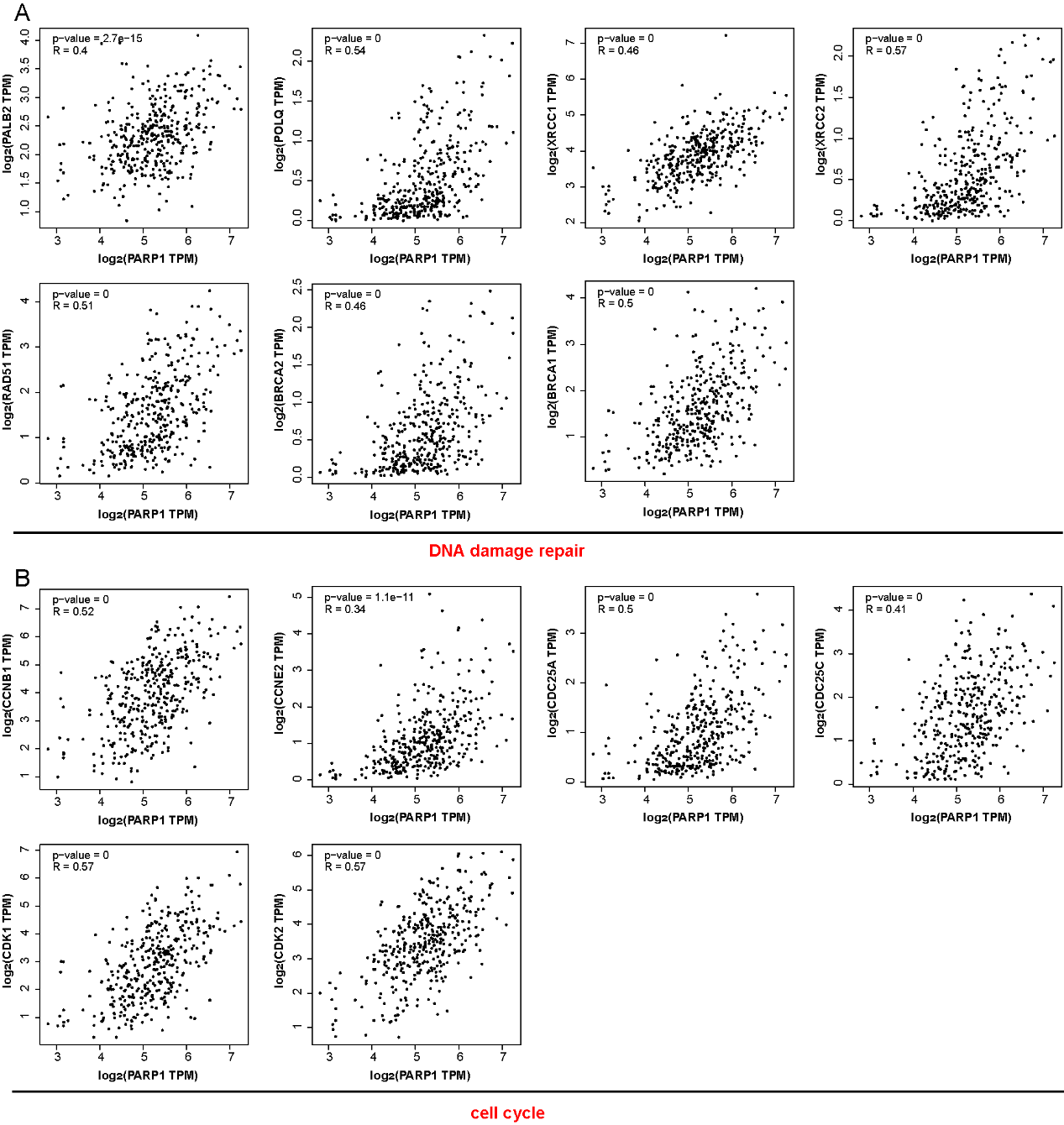


**Supplemental Figure 3. Expression correlation analysis of PARP1.** (A) Analysis of TCGA data demonstrated a significant positive correlation between PARP1 expression and DNA damage repair-related gene expression in HCC. (B) Based on the data from TCGA project, correlation analysis revealed that PAPR1 expression was positively associated with the expression of cell cycle regulation genes in HCC.
